# Surgical Management of an Intentionally Ingested Vape Device Chronically Impacted within the Duodenum of an Adult Male

**DOI:** 10.7759/cureus.39448

**Published:** 2023-05-24

**Authors:** Audra L King, David R Velez, Mentor Ahmeti

**Affiliations:** 1 Department of Surgery, University of North Dakota School of Medicine and Health Sciences, Grand Forks, USA; 2 Department of Surgery, Sanford Medical Center, Fargo, USA

**Keywords:** swallowed foreign body, late surgical intervention, surgical removal, ingested foreign body, ingested large foreign body

## Abstract

Most foreign body ingestion cases are accidental in the adult population. Intentional ingestion of foreign bodies in adults is typically associated with psychiatric disorders or developmental delay. In most cases, foreign bodies pass spontaneously through the gastrointestinal tract or can be managed endoscopically. Rarely, surgical intervention is indicated. We present a unique case of surgical management of an intentionally ingested vape device that was chronically impacted within the duodenum of an adult male present for six weeks before intervention without associated perforation. The foreign object was removed via exploratory laparotomy with duodenotomy and primary duodenorrhaphy with an uncomplicated postoperative course. There are only two previously reported cases of an ingested vape device. One was managed by observation, and the other was removed endoscopically. There are no previously reported cases of an ingested vape device that required surgical management.

## Introduction

Foreign body ingestion in the adult population is most commonly accidental. Intentional foreign body ingestion is typically associated with psychiatric conditions or developmental delay. The majority (80%) pass spontaneously within four to six days. Endoscopic intervention is required in 20% of cases, and surgical intervention is required in less than 1% of cases [1].

The majority of foreign bodies are located within the esophagus or stomach, while only 2.6% are found in the duodenum [2]. Objects with a length over 6 cm or a diameter over 2.5 cm are less likely to pass through the duodenum [1]. The duodenum contains two locations where foreign bodies may have difficult passage: the C-loop and the duodenojejunal junction[3-5]. Relative indications for surgical intervention include failure to remove endoscopically or complications that cannot be managed endoscopically. The only absolute indication for surgical intervention is perforation. Surgical consultation is often recommended if the foreign body remains impacted and does not pass through the duodenum after seven days [1].

The use of vape pens and E-cigarettes has over doubled in the last five years, particularly among youths. In a National Youth Tobacco Survey, 28% of high-school students and 11% of middle-school students had used a vape pen in the last month [6]. Of particular concern is the considerable rise in vaping among people that otherwise would never have smoked. This rise is due to the decreased stigma associated with vaping when compared to cigarette smoking.

With the rise in vape pen use, associated complications will likely become more common. We present a case of an intentionally ingested vape pen that became chronically impacted in the duodenum of an adult male for six weeks. The patient required surgical intervention for removal.

## Case presentation

**Figure 1:**
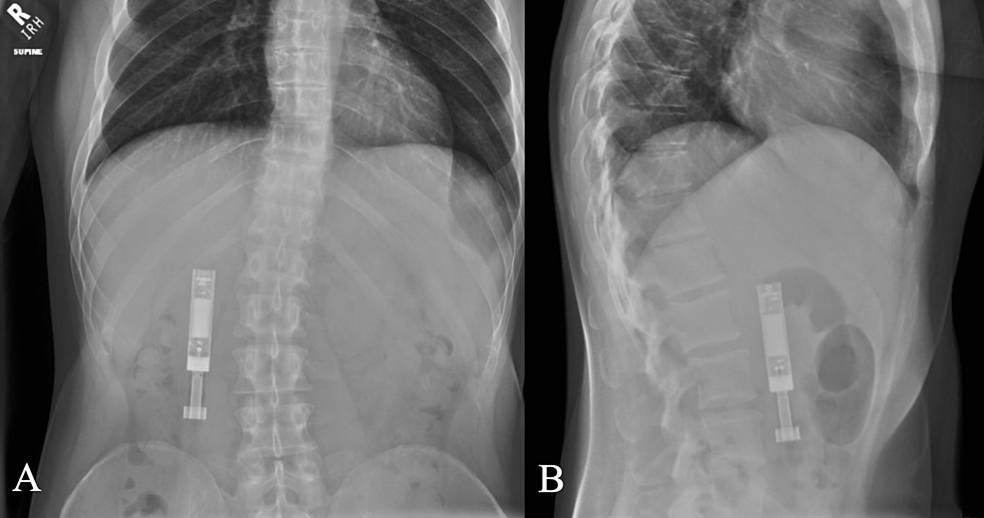
Abdominal plain film demonstrating an ingested vape pen. (A) Anterior-posterior, and (B) lateral.

A 36-year-old male with a history of seizure disorder, schizoaffective disorder, hemorrhagic stroke, chronic myelogenous leukemia, depression, anxiety, and substance abuse was initially admitted for uncontrolled acute seizures. After complaining of mild abdominal pain, nausea, and vomiting, an abdominal plain film was obtained to evaluate for ileus. Imaging saw a 10.6 cm foreign body (Figures 1A, 1B). Upon further questioning, he admitted to swallowing a vape pen six weeks prior.

Endoscopic retrieval was attempted the next day. The pen was identified in the second portion of the duodenum. It was embedded with ulceration and unable to be removed endoscopically. It was at this time that surgery was consulted. His abdominal exam was benign, with no tenderness or signs of peritonitis.

**Figure 2:**
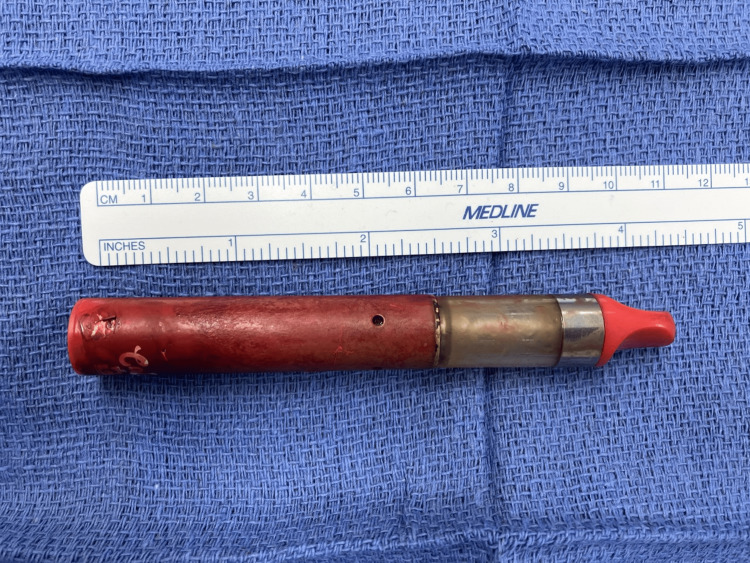
Foreign body (11.5 cm vape pen) removed at surgery.

The next day, he was taken for an exploratory laparotomy. A 2-3 cm transverse duodenotomy was made at the D1-D2 junction, and the object was easily removed (Figure 2). The duodenotomy was closed primarily in two layers. An upper GI contrast study was obtained on postoperative day one and demonstrated no evidence of a leak. His diet was gradually advanced, and his course was otherwise uncomplicated. The patient was discharged on postoperative day seven, primarily due to other medical issues.

## Discussion

After an extensive literature review, only two other cases of vape device ingestion have been reported. Meyer and Burjonrappa reported that a 16-year-old male swallowed a tetrahydrocannabinol vape cartridge after he dropped it at school and attempted to avoid search by law enforcement officers [7]. The cartridge passed on hospital day two, and he was discharged without complication. Of note, this patient had ingested only the cartridge and not the entire vape device. Lee et al. reported a 15-year-old male who swallowed an entire vape pen that was identified in his stomach [8]. The object was successfully removed endoscopically, and he otherwise progressed without complication.

Here, we present the first case report of a vape pen that required surgical management. The case is also unique in that the foreign object was present for six weeks and chronically embedded within the duodenum without any associated perforation and minimal symptoms noted by the patient. With the rise in vape pen use, associated complications such as this are likely to become more prominent.

General management of similar ingested blunt objects is as follows. Urgent endoscopic removal, within 24 hours, is required if lodged in the esophagus, although emergent removal is required for complete obstruction with an inability to handle oral secretions. Objects >5 cm long or >2 cm in diameter within the stomach or proximal duodenum should be removed by endoscopy. Smaller objects or those past the ligament of Treitz can be managed expectantly. Surgical removal is indicated for blunt objects that develop complications (obstruction or perforation) or fail to progress after one week [1]. Other objects, such as sharps, disk batteries, or magnets, may require different approaches.

## Conclusions

Similar to other foreign bodies, ingested vape pens have been treated by observation, endoscopic removal, and now surgical removal. Due to their large size, vape pens would be less likely to pass spontaneously than smaller blunt objects. At this time, the management of an ingested vape pen should follow traditional treatment algorithms as other blunt objects. This includes endoscopic intervention if the foreign object cannot pass naturally and surgical intervention if endoscopic intervention is unsuccessful or if there are signs of abdominal peritonitis.
